# New sights of spleen-preserving versus splenectomy in distal pancreatectomy for pancreatic neuroendocrine tumors: a systematic review and meta-analysis

**DOI:** 10.3389/fendo.2026.1776668

**Published:** 2026-04-16

**Authors:** Haonan Liu, Kongyuan Wei, Ruiqi Cao, Shiwei Yang, Fangzhou Wang, Shengzhan Zhang, Jiaoxing Wu, Zhengyuan Feng, Cancan Zhou, Shuai Wu, Liang Han, Zheng Wang, Qingyong Ma, Zheng Wu

**Affiliations:** 1 Department of Hepatobiliary Surgery, The First Affiliated Hospital of Xi’an Jiaotong University, Xi’an, Shaanxi, China; 2 Pancreas Center, Xi’an Jiaotong University, Xi’an, Shaanxi, China

**Keywords:** distal pancreatectomy, meta-analysis, pancreatic neuroendocrine tumors, perioperative outcomes, spleen-preservation

## Abstract

**Background:**

For resectable pancreatic neuroendocrine tumors (pNETs) located in the body or tail of pancreas that require surgery, distal pancreatectomy with splenectomy (DPS) is standard. While splenic-preserving distal pancreatectomy (SPDP) may reduce complications and provide additional benefits, its feasibility in pNETs remains uncertain. This study compares the perioperative outcomes of SPDP versus DPS for pNET patients.

**Methods:**

A comprehensive literature search was conducted in PubMed, Embase, and Web of Science included studies published before June 1, 2025. The analysis focused on primary endpoints of intraoperative blood loss (ml) and lymph nodes harvested, as well as secondary endpoints including operative time (min), transfusion, R0 resection, postoperative major complications (PMCs), postoperative pancreatic fistula (POPF), postoperative hemorrhage (PPH), hospital stay (day), lymph node metastasis (LNM), and reintervention. The pooled analysis is presented as odds ratios (OR) or mean differences (MD) with 95% confidence interval (CI). The protocol is registered on PROSPERO (CRD420251079167).

**Results:**

4 retrospective studies involving 457 patients (226 with SPDP and 231 with DPS) were analyzed. 401 patients have well-differentiated G1/G2 tumors, and the majority of SPDP patients have small tumors. Compared to DPS, SPDP had less intraoperative blood loss (SMD, -0.50, 95% CI [-0.90 to -0.11], P = 0.01), fewer lymph nodes examined (MD, -3.30, 95% CI [-5.35 to -1.24], P = 0.002), shorter operative time (MD, -31.78 min, 95% CI [-57.98 to -5.58], P = 0.02), fewer PMCs (OR, 0.57, 95% CI [0.34 to 0.95], P = 0.03) and lower transfusion rates (OR, 0.25, 95% CI [0.07 to 0.83], P = 0.02). In terms of length of hospital stay, SPDP demonstrated more favorable outcomes (MD, −1.13 days, 95% CI [−2.02 to −0.24], P = 0.01). No significant differences were observed regarding R0 resection (OR, 1.40, 95% CI [0.43 to 4.58], P = 0.58), LNM (OR, 0.95, 95% CI [0.49 to 1.85], P = 0.88), or other perioperative outcomes.

**Conclusion:**

This study proposes that SPDP may represent a feasible option for selected patients with small, well-differentiated G1/G2 pNETs, suggesting a potential role in reducing surgical risks. These findings should be interpreted as hypothesis-generating, highlighting the need for further investigation.

**Systematic review registration:**

https://www.crd.york.ac.uk/PROSPERO/recorddashboard, identifier CRD420251079167.

## Highlights

- To our knowledge, this is the first meta-analysis to explore the role of SPDP in pNETs. The pooled analysis suggests that SPDP may be a safe and effective option for managing pNETs.
- This study generates the hypothesis that for patients with small, well-differentiated G1/G2 pNETs, the application of SPDP may provide potential benefits in reducing perioperative risks.
- Further high-quality studies are warranted to refine patient selection criteria and evaluate long-term outcomes.

## Introduction

In recent years, the incidence of pancreatic neuroendocrine tumors (pNETs) has significantly increased. According to recent exploration of the Surveillance, Epidemiology and End Results (SEER) database, the incidence of pNETs is 1.00 per 100,000 individuals (1, 2). Clinically, these tumors exhibit high heterogeneity, with substantial differences in prognosis (3).

pNETs can be classified as functioning (F-pNETs) or non-functioning (NF-pNETs). Imaging by ^68^Ga/^64^Cu-DOTA-somatostatin analogue (SSA) combined with Positron Emission Tomography-Computed Tomography (PET-CT) offers high sensitivity for detecting most types of pNET lesions and should be incorporated into tumor staging, preoperative imaging and restaging (4). Several guidelines recommend that a watch-and-wait strategy as the primary approach for infrequent, small NF-pNETs, and low-grade pNETs. Tumors measuring ≥2 cm carry a higher risk of lymph node metastasis (LNM, ranging from 22% to 50%), and surgical resection is preferred (5, 6). It has been observed that low-grade tumors, measuring 1–2 cm have a lower risk of positive lymph nodes, with a 3% risk of LNM for World Health Organization grade 1 (WHO G1) tumors and up to 16% for G2 tumors (6). F-pNETs, regardless of size, should also be resected (7–9). Specifically, for patients with lesions located in the body or tail of the pancreas who meet the indications for surgical resection, distal pancreatectomy with splenectomy (DPS) and lymphadenectomy is the preferred surgical approach, utilizing either an open or minimally invasive technique (10). In certain cases (such as F-pNETs ≤ 2 cm), enucleation may be considered an alternative to standard pancreatic resection to maximally preserve native pancreatic function (11). Given the generally younger age and relatively favorable prognosis of patients with pNETs, splenic-preserving distal pancreatectomy (SPDP) may offer greater clinical benefit and is more likely to be considered in this population. Although SPDP requires a higher level of technical expertise and may limit the extent of lymphadenectomy, splenic preservation helps maintain innate immune function (12). Prior studies have indicated that patients undergoing splenectomy are at increased risk of severe septic complications, potentially resulting in a higher incidence of perioperative morbidity (13, 14). In highly malignant pancreatic diseases, such as pancreatic ductal adenocarcinoma (PDAC), SPDP has not been endorsed to avoid incomplete tumor resection, and therefore not employed (15). By contrast, the use of SPDP for premalignant lesions or low-grade malignant tumors such as pNETs remains controversial. A systematic review showed that 9%-16% of SPDP procedures were performed for pNETs (16).

A recent systematic review evaluating minimally invasive DPS and SPDP revealed that the SPDP group experienced significantly lower operative blood loss, fewer infectious complications, and reduced rates of clinically relevant postoperative pancreatic fistula (POPF) and overall complications (17). The North American Neuroendocrine Tumor Society (NANETS) Consensus indicated that preserving splenic function may confer greater benefits for low-risk pNETs (18). The European Neuroendocrine Tumor Society (ENETS) has stated that atypical resections, such as enucleation and central pancreatectomy, are acceptable for F-pNETs and well-defined small pNETs to reduce the risk of postoperative pancreatic function impairment. However, ENETS does not address the application of SPDP in this context (19). The role of SPDP in pNETs remains unclear. Some studies have identified preoperative factors to predict SPDP, such as tumor diameter <3 cm being more favorable (20). Nevertheless, the indications and feasibility of SPDP in pNETs remain contentious, with no high-level evidence currently validating the available approaches. Decisions are primarily based on the clinical experience of physicians.

With no consensus on the benefits of SPDP, the study aims to conduct a systematic review to evaluate the perioperative outcomes and oncological safety of SPDP compared to standard DPS in resectable pNETs.

## Methods

### Search stategies

This study conducted a systematic search in PubMed, Embase, and Web of Science for research related to DP in pNETs published before June 1, 2025, including both retrospective and prospective studies, utilizing the following terms: “pancreatic neuroendocrine tumors [Title/Abstract]” AND “distal pancreatectomy [Title/Abstract]”. In addition, the reference lists of selected articles were screened to assess the inclusion of other potentially relevant publications. The search strategy adhered to The PRISMA (the Preferred Reporting Items for Systematic Reviews and Meta-Analyses) guidelines (21). Detailed information regarding the search strategy is available in **Supplementary Table 1**. The specific protocol for this study can be accessed on PROSPERO (CRD420251079167).

### Study selection

Each study was independently assessed by two reviewers (LHN and WKY) based on predetermined eligibility criteria. Any discrepancies were resolved through consultation with a third reviewer (WZ) as necessary. The inclusion criteria were as follows: (1) studies involving humans; (2) randomized controlled trials (RCTs) and retrospective studies (including cohort and case-control studies) comparing SPDP with DPS; (3) in cases of duplicate publications, only the most recent and comprehensive report was included. All non-English articles, conference abstracts, letters, expert opinions, case reports, and reviews were excluded. Articles for which data could not be obtained were also excluded. The screening process is reported according to the PRISMA flow diagram (22) (**Supplementary Table 2**).

### Data extraction

Two researchers independently collected data using a standardized data abstraction form. The study summarized the characteristics of the studies and patients, primary outcomes, and other outcomes. Unless otherwise specified, the meta-analysis used mean difference (MD) and standard deviations (SD). Results reported as medians and interquartile ranges were converted into MD and SD for analysis. Data extraction included the following variables: author, year of publication, study design, study period, number of patients, age, sex, body mass index (BMI), tumor size, tumor grade, proportion of F-pNETs, proportion of minimally invasive surgeries, primary outcomes (estimated intraoperative blood loss [ml] and number of lymph nodes harvested), and additional outcomes (operating time [min], blood transfusion, complete resection [R0] margin, LNM, postoperative major complications [PMCs, Clavien-Dindo grade ≥ III (23)]), POPF, postoperative hemorrhage [PPH], length of hospital stay [day], splenic complications, and postoperative reintervention). R0 resection was defined as surgical margin greater than 1 mm. LNM was defined as positive lymph nodes demonstrated on postoperative pathological examination. POPF and PPH were defined according to the International Study Group on Pancreatic Surgery (ISGPS) (24, 25). In all included studies, “POPF A” now redefined as a “biochemical leak”, is no longer classified as a true pancreatic fistula; therefore, only POPF B and C were included in the analysis as clinically relevant POPF. All surgical complications were graded using the Clavien-Dindo classification system (26), and the grading criteria for POPF and PMCs in each article are provided in **Table 1**. Clinically relevant splenic complications defined as splenic ischemia or hemorrhage requiring reoperation for splenectomy, were also recorded.

**Table 1:** Characteristics of the studies and quality of included studies.

Published year	Sahara et al.	Huang et al.	Clément at al.	Van Beek at al.
	2021	2022	2025	2022
Design	Retrospective study	Single-center, Retrospective study	Multi-center, Retrospective study	Double-center, Retrospective study
PSM	Yes	No	Yes	No
Country	America	China	France	Netherlands
Study Period	2002-2016	2011-2021	2014-2018	2008-2019
Intervention	W-SPDP and DPS	K-SPDP and DPS	W-SPDP and DPS	SPDP and DPS
Number of participants				
Experimental	102	27	70	27
Control	102	36	70	23
Age (year)				
Experimental	59 (49–65)	48 (37–59)	59 (47–66)	56 (6 – 81)
Control	58 (47–66)	51 (38–60)	61 (54–68)	56 (18 – 76)
Sex (Males, %)				
Experimental	55 (53.9)	15 (55.6)	30 (43.0)	12 (44.4)
Control	59 (57.8)	19 (52.8)	33 (47.0)	12 (52.2)
BMI (kg/m^2^)				
Experimental	27.4 (24.0–31.2)	23.1 (21.1–25.0)	25.7 (21.5–30)	–
Control	27.4 (24.5–33.0)	23.6 (20.8–26.1)	25.8 (23.2–29.8)	–
Tumor size (cm)				
Experimental	1.6 (1.1–2.3)	1.4 (1.0–2.1)	2.5 (1.6–4.0)	1.9 (0.6, 9.3)^*^
Control	1.7 (1.2–2.3)	4.4 (2.7–6.0)	2.7 (2.5–4.5)	3.8 (0.9, 14.0)^*^
WHO Grade 1/2 (%)				
Experimental	102 (100.0)	27 (100.0)	67 (95.7)	–
Control	102 (100.0)	36 (100.0)	67 (95.7)	–
WHO Grade 3 (%)				
Experimental	0	0	1 (1.4)	–
Control	0	0	1 (1.4)	–
Functional tumor (%)				
Experimental	15 (14.7)	0	0	13 (48.1)
Control	13 (12.7)	0	0	5 (21.7)
Minimally invasive (%)				
Experimental	56 (54.9)	27 (100.0)	38 (54.3)	16 (59.3)
Control	55 (53.9)	24 (66.7)	38 (54.3)	15 (65.2)
POPF (updated ISGPS definition)	Yes	Yes	Yes	Yes
PMCs (Clavien Dindo ≥III)	Yes	Yes	Yes	Yes
Studies Quality/ Risk of Bias	Moderate	Moderate	Moderate	Moderate

PSM, propensity score matching; W-SPDP, Warshaw spleen-preserving distal pancreatectomy; K-SPDP, Kimura spleen-preserving distal pancreatectomy; DPS, distal pancreatectomy with splenectomy; BMI, Body Mass Index; WHO, World Health Organization; POPF, postoperative pancreatic fistulas; ISGPS, International Pancreatic Surgery Research Group; PMCs, postoperative major complications; *Data are presented as median [range] based on preoperative imaging.

Furthermore, different DP techniques are defined as follows: DPS involves resecting all lymph nodes along the splenic vessels, including the hilar lymph nodes. SPDP can be performed using two different methods: The Warshaw method (27), which preserves the short gastric vessels and those of the left gastric omentum while ligating the splenic arteries and veins (W-SPDP); and the Kimura method (28), which preserves both the splenic arteries and veins (K-SPDP).

### Qualitative assessment

This study utilized the Risk of Bias in Non-randomized Studies of Interventions (ROBINS-I) tool to assess the risk of bias in the included non-RCT studies (29). The overall risk of bias for each study was determined based on assessments across seven domains. For each study, two reviewers independently evaluated the risk of bias and resolved any disagreements through discussion.

Two authors (LHN and WKY) utilized the Grading of Recommendations Assessment, Development and Evaluation (GRADE) guidelines to assess the quality of the evidence (30). This evaluation was based on study design, risk of bias, inconsistency (heterogeneity), indirectness (PICO), imprecision (event numbers, confidence intervals, and sample sizes), and publication bias. Each criterion was assigned a score of “not serious,” “serious,” or “very serious.” If a criterion was rated as “serious” or “very serious”, one or two levels, respectively. The overall quality of the evidence was then categorized as high, moderate, low, or very low.

### Data synthesis and analysis

All statistical analyses were performed using Review Manager 5.4 software (The Cochrane Collaboration, Oxford, UK). We followed to the recommendations of the Cochrane Handbook for the handling of missing data. During data extraction, if missing data, such as weighted MD and SD were encountered, we calculated them following the method described in R 4.4.3 by Wan et al. (31) and using the inverse variance method. For outcomes with considerable variation in measurement scales across studies, the standardized mean difference (SMD) was used as the effect measure. If the data were unavailable, they were recorded as ‘unreported’ during the meta-analysis. For missing data that could not be obtain, only the available data were pooled for analysis, and their potential impact was assessed through subgroup or sensitivity analyses. Binary outcome variables were reported as odds ratios (OR) with corresponding 95% confidence interval (CI), computed using the Mantel-Haenszel (M-H) method. Statistical heterogeneity was assessed using the I² statistic. Given the limited number of included studies and potential clinical heterogeneity, we used the random-effects model as the primary analytical approach to provide a more conservative effect estimate (32, 33). Additionally, sensitivity analyses were conducted using the fixed-effects model to assess the robustness of the results and a leave-one-out analysis to identify potential sources of heterogeneity. Among the four included studies, two (34, 35) employed propensity score matching (PSM) in their original analyses, and only post-matching data were extracted for the pooled analysis, as matched cohorts provide better control for confounding and yield more reliable effect estimates in observational studies. These PSM studies were combined directly with non-PSM studies in the primary meta-analysis, as all studies reported comparable outcome measures (36, 37). To assess whether differences in study design (PSM vs. non-PSM) introduced confounding bias and influenced the pooled estimates, we performed a subgroup analysis stratifying by PSM use. When meta-analysis was not appropriate, data were described descriptively. P-values < 0.05 were considered statistically significant, and 95% CIs were calculated for efficacy measures.

## Results

### Search results

**Figure 1:**
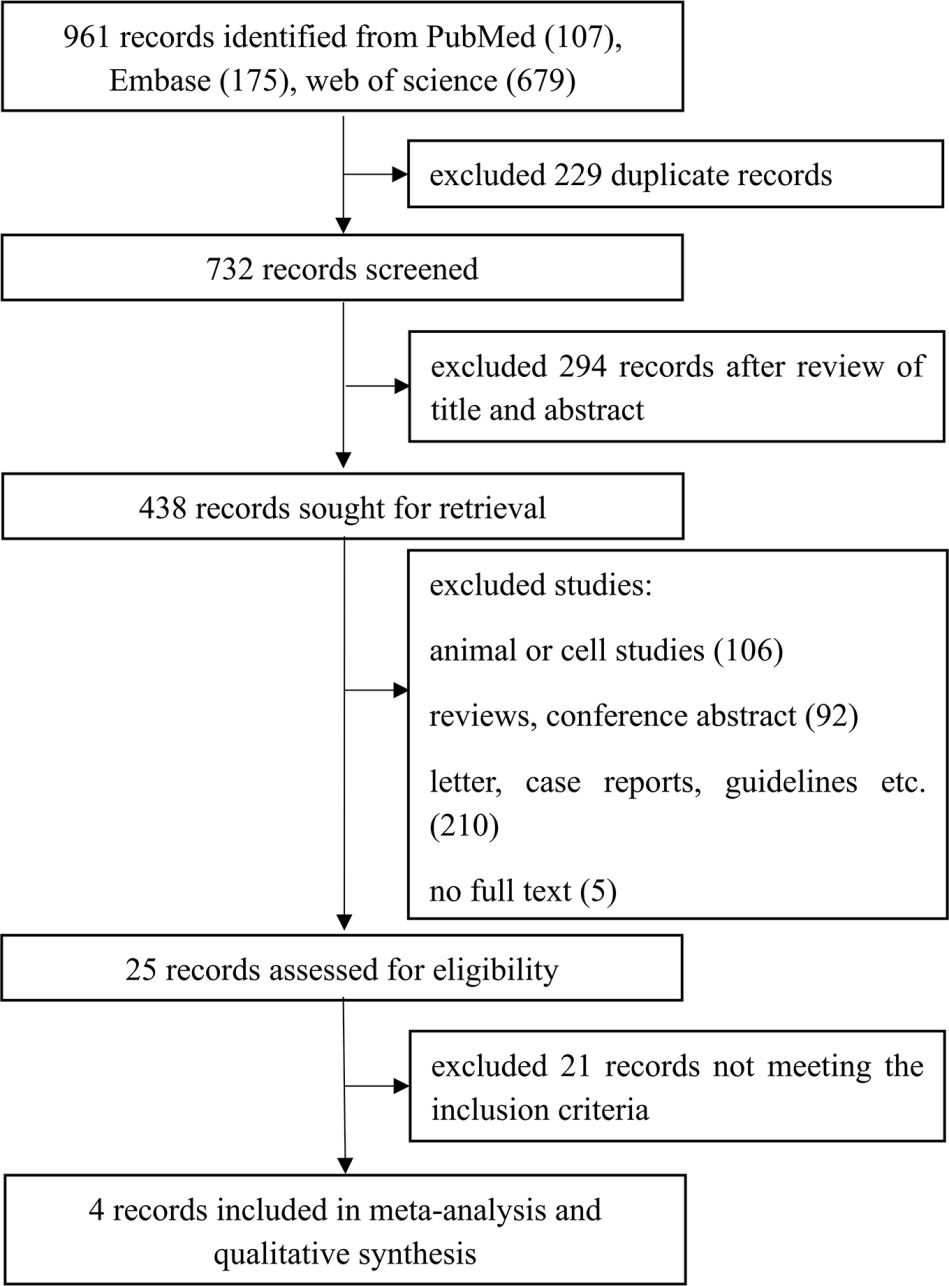
An overview of literature search and selection process.

The initial search identified 961 articles, of which 732 remained after removing 229 duplicates. Following a review of titles and abstracts, 294 articles were excluded, and an additional 413 were excluded based on predefined criteria (reviews, letters, animal studies, guidelines, non-English articles, case reports, and studies without control groups). Full-text reviews were conducted for 25 articles to assess compliance with inclusion criteria. Ultimately, 4 studies met all inclusion criteria and were included in this meta-analysis (**Figure 1**).

### Characteristics of included studies

**Table 1** presents the baseline characteristics of the studies and patients. Four retrospective studies met the inclusion criteria. One study utilized cases from the US Neuroendocrine Tumor Study Group (US-NETSG) database (35), while the other three (34, 38, 39) were included in our systematic review and meta-analysis as single-, double-, and multi-center retrospective studies, respectively. All four studies reported relevant perioperative outcomes, collectively encompassing 457 patients. All patients had histologically confirmed pNETs and were eligible for radical DP; two studies (35, 39) exclusively included NF-pNETs. Among 407 patients, 401 (98.5%) had G1/G2 tumors, while Van Beek’s study didn’t report tumor grade. The studies by Clement (34), Huang (38), and Van Beek (39) included patients who underwent various preoperative imaging modalities, such as systematic chest and abdominal CT, magnetic resonance imaging (MRI), or ^68^Ga-DOTATATE PET/CT scans to detect regional and distant metastases; this aspect was not detailed in Sahara’s study (35). Two studies (34, 35) performed PSM, and data after PSM were used for the meta-analysis, as this approach provides more reliable estimates by balancing potential confounders between groups. Notably, the surgical procedures in these two studies (34, 35) employed W-SPDP, whereas Huang et al. (38) used K-SPDP. Overall, 254 patients (55.6%) underwent minimally invasive surgical approaches, with specific proportions detailed in **Table 1**.

### Quality assessment of the included studies

The literature included in this study comprised 4 non-RCTs (34, 35, 38, 39). These studies were assessed using the ROBINS-I tool and rated as moderate-risk. (**Table 1**).

### Meta-analysis

#### Primary outcomes

**Figure 2:**
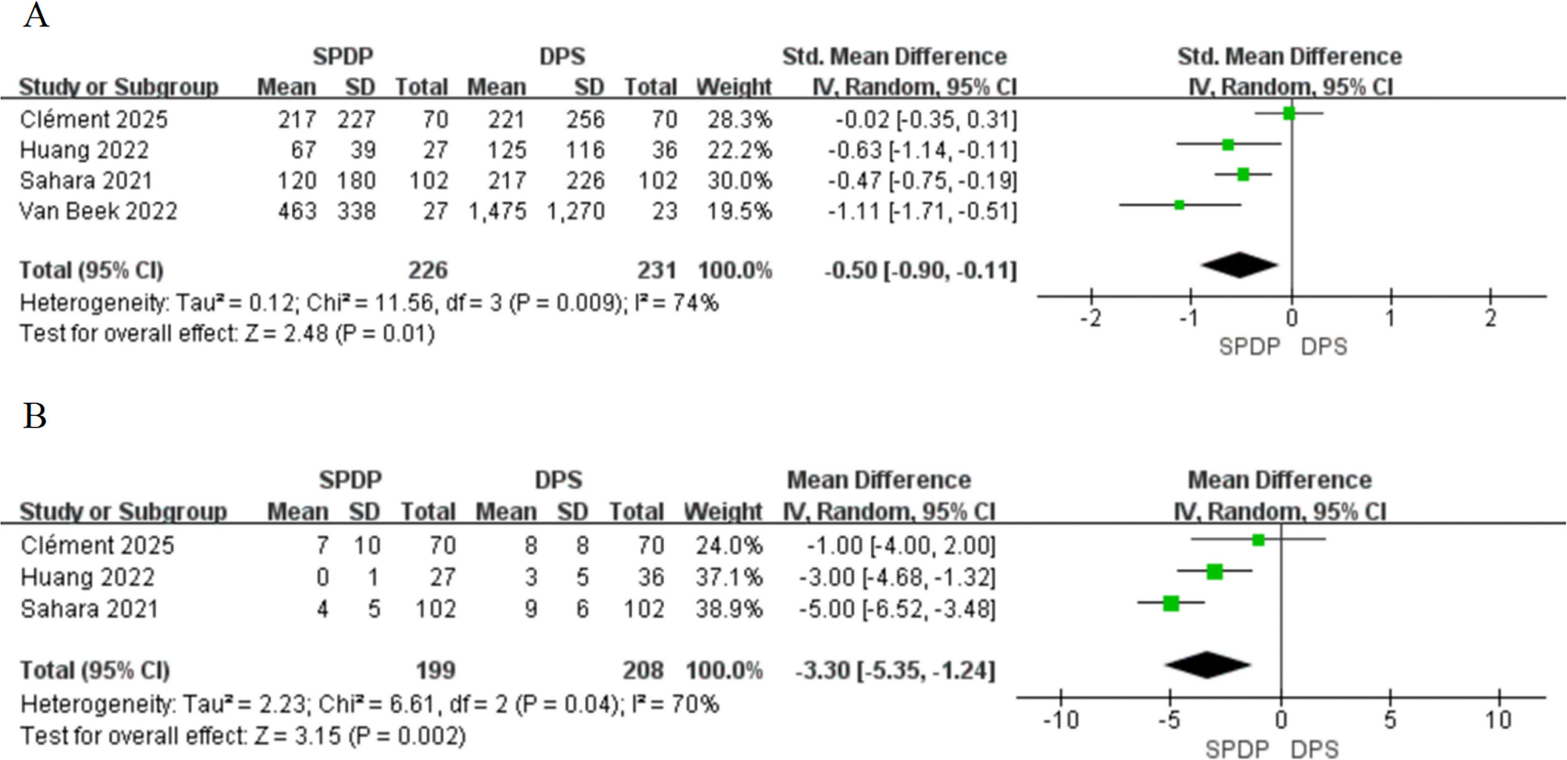
Forest plot showing the outcome of operative factors among patients undergoing splenic-preserving distal pancreatectomy (SPDP) and distal pancreatectomy with splenectomy (DPS): **(A)** Estimated blood loss (ml); **(B)** Number of lymph nodes examined.

Three (35, 38, 39) of the four studies indicated that SPDP significantly reduced intraoperative blood loss. The overall summary analysis (**Figure 2A**) supported this conclusion, demonstrating that patients in the SPDP group had lower estimated intraoperative blood loss (SMD, -0.50, 95% CI [-0.90 to -0.11], P = 0.01). Moderate heterogeneity was observed among the outcomes (I² = 74%, P = 0.009). Regarding intraoperative lymphadenectomy, three studies (34, 35, 38) (457 participants; **Figure 2B**) reported the number of lymph nodes removed, showing that the number of lymph nodes harvested in the SPDP group was significantly lower than in the DPS group (MD, -3.30, 95% CI [-5.35 to -1.24], P = 0.002), with a statistically significant difference and high heterogeneity among the results (I^2^ = 70%, P = 0.04).

#### Additional outcomes

**Figure 3:**
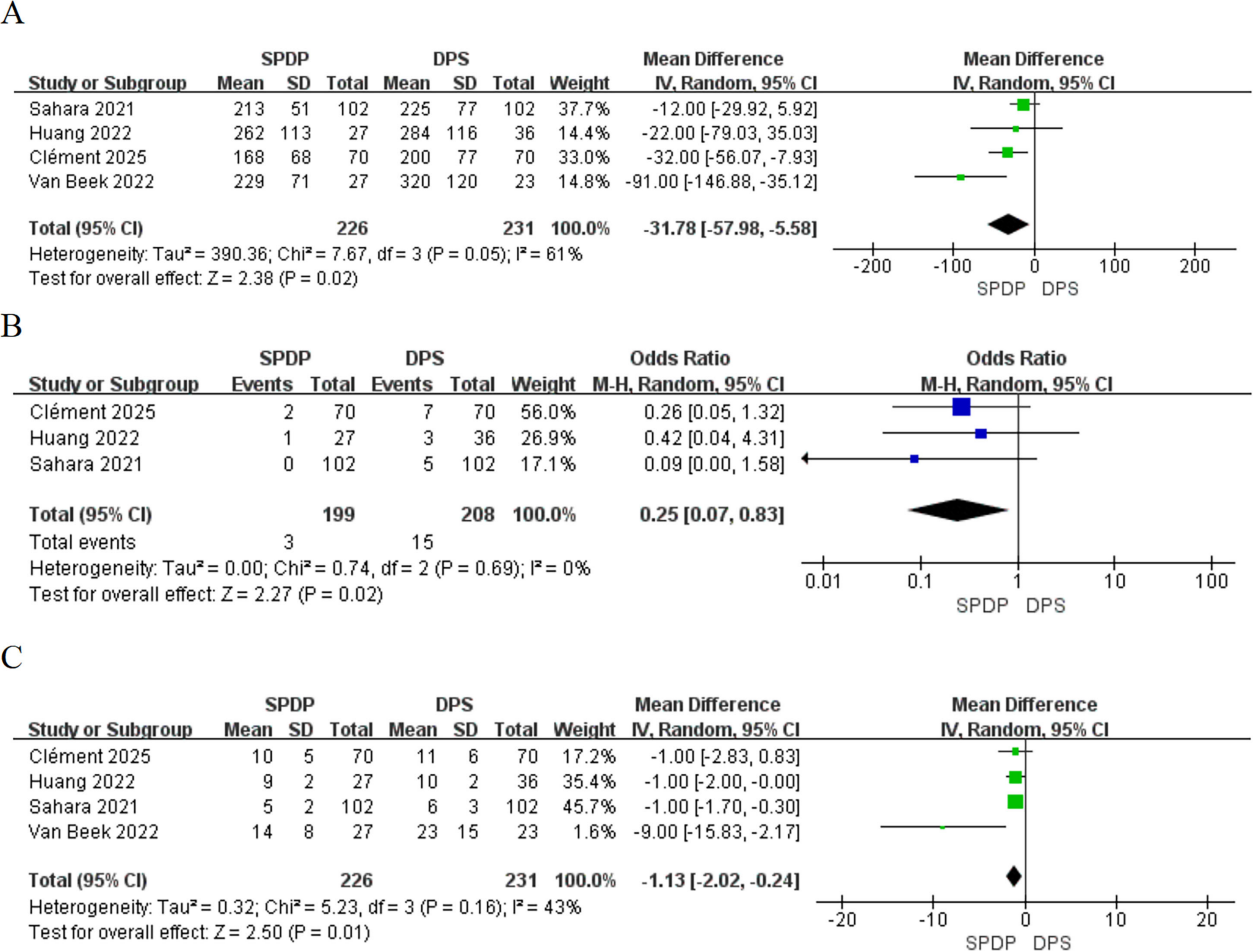
Forest plot showing the outcome among patients undergoing splenic-preserving distal pancreatectomy (SPDP) and distal pancreatectomy with splenectomy (DPS): **(A)** Operation time (min); **(B)** Blood transfusion; **(C)** Hospital stay (days).

All studies (457 participants) reported intraoperative outcome comparisons between SPDP and DPS (34, 35, 38, 39). The pooled results demonstrated that SPDP significantly reduced operative time compared to DPS, with a statistically significant difference (MD, -31.78 min, 95% CI [-57.98 to -5.58], P = 0.02). The results of the meta-analysis are illustrated in **Figure 3A**. The heterogeneity among the combined results was moderate (I² = 61%, P = 0.05). An analysis of transfusion rates across the three studies indicated that the SPDP group required significantly fewer transfusions than the DPS group, with a statistically significant difference (OR, 0.25, 95% CI [0.07 to 0.83], P = 0.02). The results were homogeneous (I^2^ = 0%, P = 0.69). (**Figure 3B**). All studies (34, 35, 38, 39) evaluated postoperative length of hospital stay, and the pooled analysis revealed that SPDP significantly shortened hospital stays compared to DPS (MD, −1.13 days, 95% CI [−2.02 to −0.24], P = 0.01) (**Figure 3C**).

**Figure 4:**
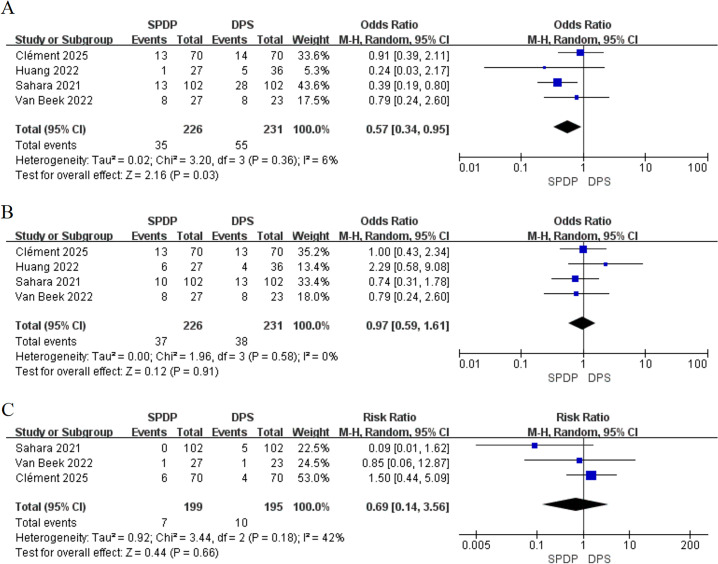
Forest plot showing the postoperative outcomes among patients undergoing splenic-preserving distal pancreatectomy (SPDP) and distal pancreatectomy with splenectomy (DPS): **(A)** Postoperative major complications (PMCs); **(B)** clinically relevant post-operative pancreatic fistula (POPF); **(C)** post-pancreatectomy hemorrhage (PPH) after the surgery.

Four studies (34, 35, 38, 39) (457 patients) reported on PMCs (**Figure 4A**), and the findings indicated a lower incidence of PMCs in the SPDP group (OR, 0.57, 95% CI [0.34 to 0.95], P = 0.03). The results showed low heterogeneity (I^2^ = 6%, P = 0.36). Four studies (34, 35, 38, 39) also reported on the occurrence of POPF (457 patients; **Figure 4B**), revealing no significant difference (OR, 0.97, 95% CI [0.59 to 1.61], P > 0.05). These results were homogeneous (I^2^ = 0%, P = 0.58). Three studies (34, 35) reported on PPH (394 patients; **Figure 4C**), with no statistically significant difference observed between the groups (OR, 0.69, 95% CI [0.14 to 3.56], P > 0.05). The results exhibited low heterogeneity (I^2^ = 42%, P = 0.18).

**Figure 5:**
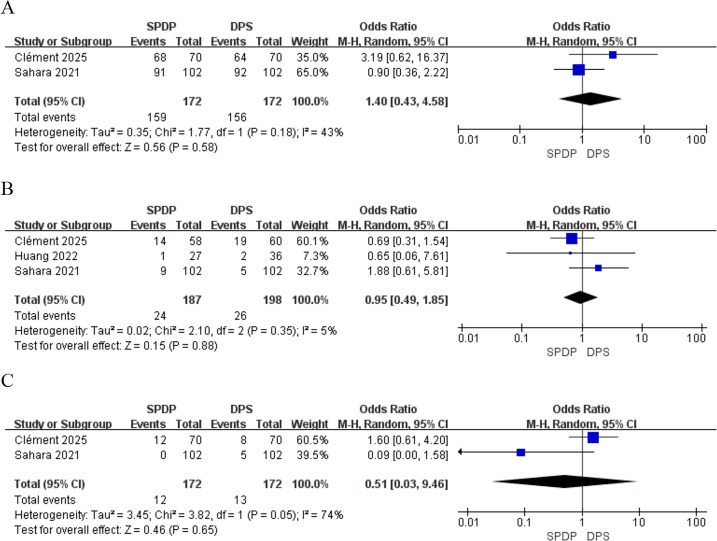
Forest plot showing the postoperative outcomes among patients undergoing splenic-preserving distal pancreatectomy (SPDP) and distal pancreatectomy with splenectomy (DPS): **(A)** R0 margin status; **(B)** Lymph node metastasis (LNM); C.Reintervention.

Additionally, only two studies (34, 35) (344 participants; **Figure 5A**) reported the R0 resection rates, indicating that SPDP and DPS achieve comparable R0 resection rates (OR, 1.40, 95% CI [0.43 to 4.58], P = 0.58), with low heterogeneity (I^2^ = 43%, P = 0.18). Three studies reported LNM (385 patients; **Figure 5B**), demonstrating no significant difference (OR, 0.95, 95% CI [0.49 to 1.85], P = 0.88). Two studies (34, 35) (344 patients; **Figure 5C**) assessed the occurrence of reintervention, with the summary results showing no significant difference in reintervention rates between the two groups (OR = 0.51, 95% CI [0.03 to 9.46], P > 0.05).

Three studies reported spleen-specific clinical outcomes; however, because these were isolated cases, a pooled statistical analysis was not feasible. In Clement’s study, there was one splenic complication (1.4%) in the W-SPDP group, which required rescue splenectomy on postoperative day 11 due to concomitant POPF and PPH. In the study by Sahara, sepsis following surgery was observed in 1 SPDP patient (1.0%) and 3 DPS patients (2.9%). Patients undergoing SPDP had a lower incidence of deep surgical site infections (1 patient, 1.0% vs. 9 patients, 8.8%). Deep venous thrombosis was noted in 1 SPDP patient (1.0%) and 3 DPS patients (2.9%), while pulmonary embolism affected 2 SPDP patients (2.0%) and 3 DPS patients (2.9%). Van Beek’s study (39) reported that no patient experienced thrombosis, pulmonary embolism or arterial thrombosis. However, 4 patients (14.8%) in the SPDP group were diagnosed with splenic infarction/ischemia. Infections (pulmonary, urologic, and catheter-related) occurred in 4 patients (14.8%) in the SPDP group and 3 patients (13.0%) in the DPS group.

### Subgroup analysis

To further investigate the impact of study design on the outcomes, we conducted subgroup analyses based on the use of PSM. For the primary outcome measures (estimated blood loss and number of lymph nodes dissected), the difference between the groups was not statistically significant when only two the PSM studies were pooled, and heterogeneity among studies was substantially reduced (**Supplementary Figure 1**). However, the overall pooled analysis, which included non-PSM studies, revealed a significant difference between groups. The subgroup analysis of the PMCs also revealed such differences. Notably, the forest plot showed that the direction of the effect sizes across studies was generally consistent, suggesting that non-PSM studies may have overestimated effect sizes. This indicates that confounding bias introduced by study design differences influenced the overall conclusions. Therefore, the positive findings for the primary outcomes should be interpreted with caution, and potential biases arising from variations in original study designs must be carefully considered. For other outcome measures (operative time, blood transfusion rate, hospital stay, POPF, PPH and LNM), the results of the subgroup analyses were consistent with the overall pooled analysis, which supports the robustness of the study conclusions to some extent (**Supplementary Figures 2**-**5**).

Two studies included in this analysis performed PSM. To explore the potential impact of PSM on the pooled estimates, we also incorporated the available pre-matching data into the analysis, as shown in **Supplementary Figures 6**-**8**. However, it should be noted that one study (35) reported only baseline characteristics of the pre-matching cohorts and did not provide pre-matching outcome data; consequently, we combined only the pre-matching data from the other study with the remaining data for analysis (34). The results indicated that for most outcome measures, the direction and magnitude of effect sizes were consistent with the main analysis using PSM data. The transfusion rate and the incidence of PMCs no longer showed statistically significant differences when pre-matching data were included (P > 0.05). This may reflect the effect of PSM in balancing confounding factors, as the pre-matching cohorts likely contained a greater degree of baseline imbalance and selection bias.

### Sensitivity analysis

Sensitivity analyses were performed using a leave-one-out approach for outcomes with moderate heterogeneity (estimated blood loss and the number of intraoperative lymph nodes harvested) to assess the stability of the results. The overall effect trend remained consistent regardless of which single study was excluded. The heterogeneity observed in our analysis may be attributed to the relatively small sample size in the two non-PSM studies. In addition, sensitivity analyses using fixed-effects models yielded results consistent with the primary random-effects analyses for all outcomes, supporting the robustness of our findings.

### Publication bias

Due to the limited number of included studies, publication bias could not be reliably assessed.

### Quality of the evidence

GRADE was applied to the outcomes supported by the meta-analysis, including intraoperative blood loss, number of lymph nodes removed during surgery, blood transfusion rates, operative time, length of hospital stay, and PMCs. According to the GRADE assessment, the quality of evidence for intraoperative blood loss, operative time, blood transfusion rates, length of hospital stay, and PMCs was rated as low, while the overall quality of evidence for the number of lymph nodes removed during surgery was rated as very low (**Table 2**). The primary reasons for this assessment included the limited number of studies analyzed, all of which were non-RCTs. Specific factors contributing to this evaluation are further detailed in **Table 2**.

**Table 2:** Certainty of evidence assessment using the GRADE (Grading of Recommendations Assessment, Development and Evaluation) approach.

Factor	Intraoperative blood loss	Number of lymph nodes examined	Operative time	Blood transfusion	Length of hospital stay	PMCs
No. of studies	4	3	4	3	4	4
Study design	Non-RCT	Non-RCT	Non-RCT	Non-RCT	Non-RCT	Non-RCT
Certainty assessment						
Risk of bias	Not serious^a^	Not serious^a^	Not serious^a^	Not serious^a^	Not serious^a^	Not serious^a^
Inconsistency	Not serious	Serious^b^	Not serious	Not serious	Not serious	Not serious
Indirectness	Not serious	Not serious	Not serious	Not serious	Not serious	Not serious
Imprecision	Not serious	Not serious	Not serious	Not serious	Not serious	Not serious
publication bias	Undetected^c^	Undetected^c^	Undetected^c^	Undetected^c^	Undetected^c^	Undetected^c^
Other considerations	None	None	None	None	None	None
No. of patients	457	407	457	407	457	457
Risk difference	SMD, 0.50 lower, 95% CI (0.90 lower to 0.11 lower)	MD, 3.30 lower, 95% CI (5.35 lower to 1.24 lower)	MD, 31.78 lower, 95% CI (57.98 lower to 5.58 lower)	OR, 0.25 higher, 95% CI (0.07 higher to 0.83 higher)	MD, 1.13 lower, 95% CI (2.02 lower to 0.24 lower)	OR, 0.57, higher, 95% CI (0.34 higher to 0.95 higher)
Certainty	○○○○ Low	⊕○○○ Very Low	○○○○ Low	○○○○ Low	○○○○ Low	○○○○ Low

RCT, randomized controlled trial; MD, mean difference; CI, confidence interval; PMCs, postoperative major complications. ^a^ All three studies were non-RCTs, but two of these studies performed strict propensity score matching. No adjustment. ^b^ Serious inconsistency since I^2^ = 85%. Downgraded. ^c^ Not enough trials to judge.

## Discussion

Although numerous studies have reported on SPDP for various pancreatic diseases, only four retrospective studies have specifically focused on pNETs, yielding inconsistent conclusions regarding its impact on outcomes. To date, no systematic review has evaluated the outcomes of SPDP for pNETs. Based on the results of this study, SPDP was found to reduce operative time, intraoperative blood loss, PMCs and transfusion rates compared to DPS. Additionally, SPDP was associated with fewer lymph nodes harvested while achieving comparable rates of R0 resection and LNM. Although no significant advantages were observed in postoperative complications like POPF and PPH, SPDP significantly shortened the length of hospital stay. These findings preliminarily propose the safety and efficacy of SPDP for resectable pNETs. However, subgroup analyses based on PSM revealed important nuances. For the primary outcomes of estimated blood loss and number of lymph nodes dissected, pooling only the two PSM studies yielded non-significant differences and substantially reduced heterogeneity, whereas the overall pooled analysis (including non-PSM studies) showed significant benefits. Similar patterns were observed for PMCs. Although the direction of effect sizes was consistent across studies, these findings suggest that non-PSM studies may have overestimated the treatment effects, and that confounding bias inherent in the original study designs could influence the overall conclusions. For other outcomes, subgroup analyses were consistent with the main analysis, supporting the robustness of these findings to some extent. Overall, these findings preliminarily suggest potential benefits and emerging trends of SPDP for resectable pNETs, but given the low to very low quality of evidence (all non-RCT studies). The positive findings should be interpreted with caution. This underscores the need for future RCTs to confirm these findings and provide higher-level evidence.

Lymph node dissection remains a controversial aspect of pNET management. The risk of LNM necessitates the recommendation for regional lymph node dissection during resection. While some studies have confirmed the prognostic significance of total lymph node examination and LNM in pNETs, others suggest that in tumors with favorable features, such as a tumor size of 2 cm or a Ki-67 index < 3%, lymph node examination and LNM may have limited prognostic value. Previous research supports minimally invasive approaches for small pNETs measuring less than 2 cm, as well as parenchyma-sparing pancreatic resections or avoidance of lymphadenectomy when feasible (40). This recommendation is based on the indolent biology and the very low risk of local recurrence. Current guidelines remain controversial regarding the optimal extent of lymphadenectomy in pNET surgery; however, all recommend tumor resection with lymph node dissection when imaging reveals suspicious lymph nodes (4, 18). The present study primarily involved G1/G2 tumors, suggesting that the actual likelihood of LNM may be very low. The meta-analysis showed that although the number of lymph node dissected was significantly lower in the SPDP group compared to the DPS group (MD -3.30; 95% CI [-5.35 to -1.24]; P = 0.002), raising concerns about the adequacy of tumor clearance, the proportion of positive lymph nodes did not exhibit a significant difference (OR, 0.95; 95% CI [0.49 to 1.85]; P = 0.88). It has been reported that lymph node dissection in patients undergoing resection for pNETs of the pancreatic body or tail does not confer additional therapeutic benefit, consistent with our findings. It appears that the indication for lymph node dissection in well-differentiated pNETs warrants further investigation. Subgroup analysis revealed no significant difference in the number of lymph node dissected, possibly due to the heterogeneity introduced by studies not employing PSM. Nevertheless, these findings offer valuable insights for future research in this area.

Three studies reported survival curves for long-term outcomes; however, a combined analysis was precluded due to the heterogeneity and incompleteness of the data. Nevertheless, SPDP did not appear to increase the risk of adverse long-term outcomes. In Clement’s study (34), overall survival (OS) in the SPDP group did not reach statistical significance; however, the 5-year recurrence-free survival (RFS) was significantly shorter (not reached vs. 59 months; P = 0.028), which may be related to the potential immune preservation in the W-SPDP group. Huang’s (38) and Sahara’s study (35) yielded no difference in 5-year OS and RFS between groups. These findings suggest that the surgical approach does not influence the long-term outcomes of patients with pNETs. Spleen preservation may therefore be a feasible option when technically appropriate.

As a critical immune organ, the spleen plays a vital role, and splenectomy increases the risk of both short- and long-term infectious complications, as well as portal vein thrombosis, thrombocytosis, and hypercoagulability. Previous studies on other pancreatic diseases have established the non-inferiority of SPDP, highlighting advantages such as improved perioperative outcomes and preservation of splenic function. Gorris et al. (41) conducted a large international multicenter retrospective study involving patients with intraductal papillary mucinous neoplasms (IPMNs) undergoing SPDP and DPS. Their findings indicated that SPDP was associated with shorter operative times, reduced blood loss, and shorter hospital stays, consistent with the trends observed in the present study. Supporting these results, systematic reviews and meta-analyses by Shi et al. (13) and He et al. (42) in patients with benign and low-grade malignant pancreatic tumors confirmed that SPDP significantly reduces postoperative complications and intraoperative blood loss. Additionally, He et al. reported shorter hospital stays and fewer intra-abdominal abscesses, while no significant difference was observed in other outcomes such as operative time.

Although the present study reached similar conclusions, its limited sample size precluded further stratified analyses by surgical approach or technique. A preliminary review of the original data indicated that SPDP is more frequently performed using minimally invasive techniques, which may explain the observed improvements in intraoperative outcomes. Previous PSM studies on benign and low-grade malignant pancreatic lesions have shown that, although technically more challenging, laparoscopic SPDP is associated with significantly shorter operative time than DPS. Van Beek’s study did not report the specific approach of SPDP. The majority of reported cases included in this study were performed using the W-SPDP (172 cases), whereas only 27 cases involved the K-SPDP. An international multicenter retrospective study demonstrated that W-SPDP was associated with less blood loss and a trend towards shorter surgical time compared to K-SPDP, though both techniques were deemed safe options (43). These findings suggest a preference for W-SPDP in pNETs, but further comparative cohort studies are needed to determine the optimal technique.

Notably, the generalizability of our findings should be approached with caution due to limitations inherent in the included literature. Most tumors analyzed were well-differentiated G1/G2 and NF-pNETs, suggesting that our conclusions may be particularly relevant to this tumor subtype. Patients with suspected direct tumor involvement or close relationship with the spleen or splenic vessels based on preoperative or intraoperative findings were reported to be excluded from the SPDP group in several research. Regarding tumor size, three of the included studies (35, 38) preferentially selected smaller tumors for SPDP, implying that our findings may be more applicable to small pNETs. This aligns with previous evidence indicating that a tumor size <3 cm is a favorable preoperative predictor for spleen preservation in benign and low-grade malignant pancreatic tumors (20). Although Clément et al. (35, 38) suggested that SPDP may not have a definitive size limitation in pNETs—noting that K-SPDP was primarily performed for tumors <2 cm while W-SPDP and DPS were reserved for more advanced tumors (>2 cm)—and other authors have similarly proposed that SPDP could be applied without strict size restrictions, we interpret these findings with caution. The current evidence remains insufficient, largely based on single-center experiences, and further studies are warranted to define the indications for SPDP in pNETs. Therefore, our study offers preliminary hypotheses and reflections rather than definitive recommendations.

In this study, we acknowledge several limitations. First, only four non-RCTs were identified, reflecting the limited research on SPDP in pNETs. According to the ROBINS-I assessment, the quality of these studies ranged from low to moderate, which may introduce selection bias and limit evidence strength. Second, two of the included studies employed PSM, while the other two did not. Although we extracted post-matching data from the PSM studies to minimize confounding, combining PSM and non-PSM studies in a single meta-analysis remains methodologically challenging, as design-related differences may introduce bias and affect comparability. Additionally, due to restriction in the original data, we were unable to obtain key outcome indicators such as postoperative infection, sepsis, splenic infarction, and long-term outcomes, preventing a comprehensive analysis. We also did not perform stratified analyses based on the surgical approach (open versus minimally invasive) or surgical techniques (W-SPDP and K-SPDP), which likely contributed to heterogeneity in the pooled outcomes. Nevertheless, this study preliminarily concludes that SPDP may be safe and effective for resectable, small, well-differentiated G1/G2 pNETs. Large-scale RCTs with higher levels of evidence are needed for further validation.

## Conclusion

To our knowledge, this is the first meta-analysis comparing the surgical outcomes of SPDP and DPS for pNETs. The findings suggest that SPDP may be a safe and feasible option for selected patients with resectable, well-differentiated G1/G2, small pNETs, and may offer potential benefits in reducing certain surgical risks. However, the quality of evidence supporting these positive outcomes was rated as low to very low. Well-designed clinical trials are warranted in the future to further evaluate the role of SPDP and its long-term outcomes in specific patient populations.

## Data Availability

The datasets presented in this study can be found in online repositories. The names of the repository/repositories and accession number(s) can be found in the article/**Supplementary Material**.

## Supplementary material

Supplementary Data Sheet 1 The checklist in accordance with the PRISMA.

Supplementary Figure 1 Clinical outcomes of patients undergoing splenic-preserving distal pancreatectomy (SPDP) and distal pancreatectomy with splenectomy (DPS), categorized into subgroups according to the application of propensity score matching (PSM): **(A)** Estimated blood loss (ml); **(B)** Number of lymph nodes examined.

Supplementary Figure 2 Clinical outcomes of patients undergoing splenic-preserving distal pancreatectomy (SPDP) and distal pancreatectomy with splenectomy (DPS), categorized into subgroups according to the application of propensity score matching (PSM): **(A)** Operation time; **(B)** Blood transfusion.

Supplementary Figure 3 Clinical outcomes of patients undergoing splenic-preserving distal pancreatectomy (SPDP) and distal pancreatectomy with splenectomy (DPS), categorized into subgroups according to the application of propensity score matching (PSM): **(A)** Hospital stay (days); **(B)** Major complications.

Supplementary Figure 4 Clinical outcomes of patients undergoing splenic-preserving distal pancreatectomy (SPDP) and distal pancreatectomy with splenectomy (DPS), categorized into subgroups according to the application of propensity score matching (PSM): **(A)** Clinically relevant post-operative pancreatic fistula (POPF); **(B)** post-pancreatectomy hemorrhage (PPH) after the surgery.

Supplementary Figure 5 Clinical outcomes of patients undergoing splenic-preserving distal pancreatectomy (SPDP) and distal pancreatectomy with splenectomy (DPS), categorized into subgroups according to the application of propensity score matching (PSM): Lymph node metastasis.

Supplementary Figure 6 Clinical outcomes of patients undergoing splenic-preserving distal pancreatectomy (SPDP) and distal pancreatectomy with splenectomy (DPS) based on data before propensity score matching: **(A)** Estimated blood loss (ml); **(B)** Number of lymph nodes examined; **(C)** Lymph node metastasis (LNM).

Supplementary Figure 7 Clinical outcomes of patients undergoing splenic-preserving distal pancreatectomy (SPDP) and distal pancreatectomy with splenectomy (DPS) based on data before propensity score matching: **(A)** Operation time (min); **(B)** Blood transfusion; **(C)** Hospital stay (day).

Supplementary Figure 8 Clinical outcomes of patients undergoing splenic-preserving distal pancreatectomy (SPDP) and distal pancreatectomy with splenectomy (DPS) based on data before propensity score matching: **(A)** Postoperative major complications (PMCs); **(B)** clinically relevant post-operative pancreatic fistula (POPF); **(C)** post-pancreatectomy hemorrhage (PPH) after the surgery.
