# Supplementary material for: New sights of spleen-preserving versus splenectomy in distal pancreatectomy for pancreatic neuroendocrine tumors: a systematic review and meta-analysis
Source: Front Endocrinol (Lausanne). 2026 Apr 16;17:1776668. doi: 10.3389/fendo.2026.1776668 (PMC13129563; doi:10.3389/fendo.2026.1776668)
Supplement: Supplementary Data Sheet 1 — The checklist in accordance with the PRISMA. [file Table1.docx]

**Supplementary Table 1.** Search strategies.

| **PubMed** | |
| --- | --- |
| #3 | #1 AND #2 |
| #2 | "pancreatic neuroendocrine tumors" [title/abstract] |
| #1 | "distal pancreatectomy" [title/abstract] |
| **Embase** | |
| #3 | #1 AND #2 |
| #2 | ‘pancreatic neuroendocrine tumors’ |
| #1 | ‘distal pancreatectomy’ |
| **Web of Science** | |
| #1 | TS= ("distal pancreatectomy") |
| #2 | TS= ("pancreatic neuroendocrine tumors") |
| #3 | #1 AND #2 |
