# Supplementary figures and images for: New sights of spleen-preserving versus splenectomy in distal pancreatectomy for pancreatic neuroendocrine tumors: a systematic review and meta-analysis
Source: Front Endocrinol (Lausanne). 2026 Apr 16;17:1776668. doi: 10.3389/fendo.2026.1776668 (PMC13129563; doi:10.3389/fendo.2026.1776668)

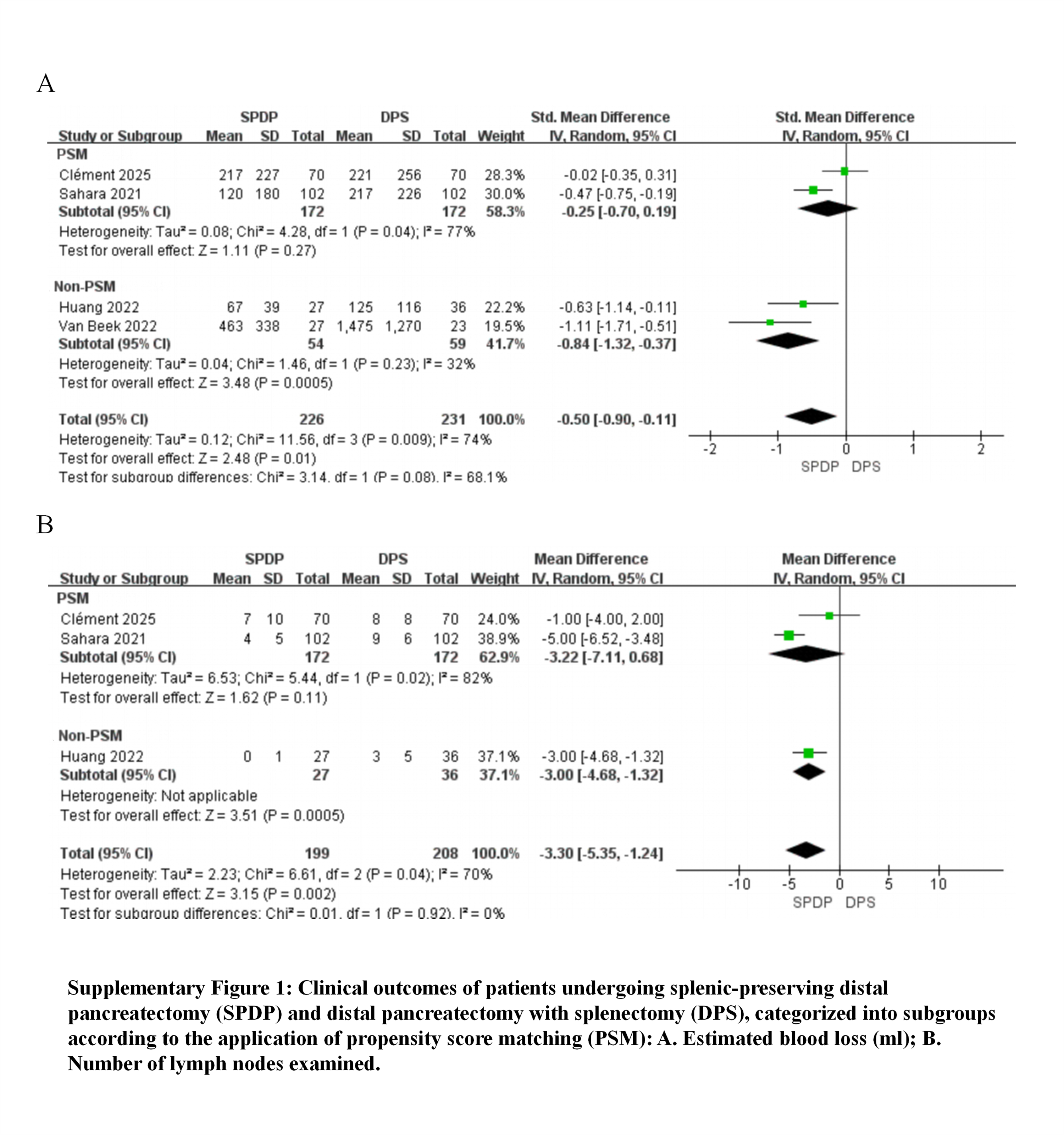

Supplement: Supplementary Figure 1 — Clinical outcomes of patients undergoing splenic-preserving distal pancreatectomy (SPDP) and distal pancreatectomy with splenectomy (DPS), categorized into subgroups according to the application of propensity score matching (PSM): (A) Estimated blood loss (ml); (B) Number of lymph nodes examined. [file Image1.tif]

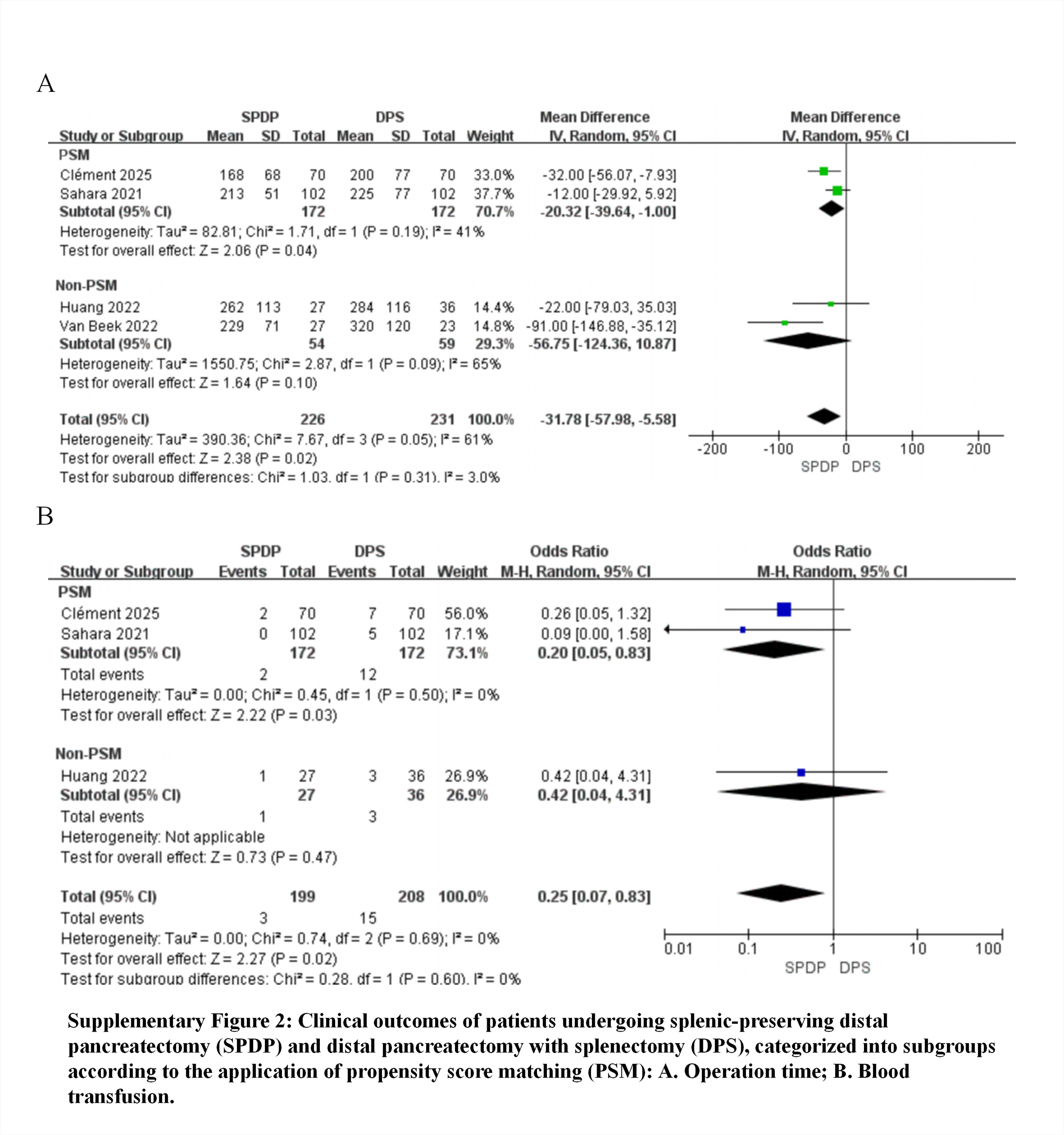

Supplement: Supplementary Figure 2 — Clinical outcomes of patients undergoing splenic-preserving distal pancreatectomy (SPDP) and distal pancreatectomy with splenectomy (DPS), categorized into subgroups according to the application of propensity score matching (PSM): (A) Operation time; (B) Blood transfusion. [file Image2.tif]

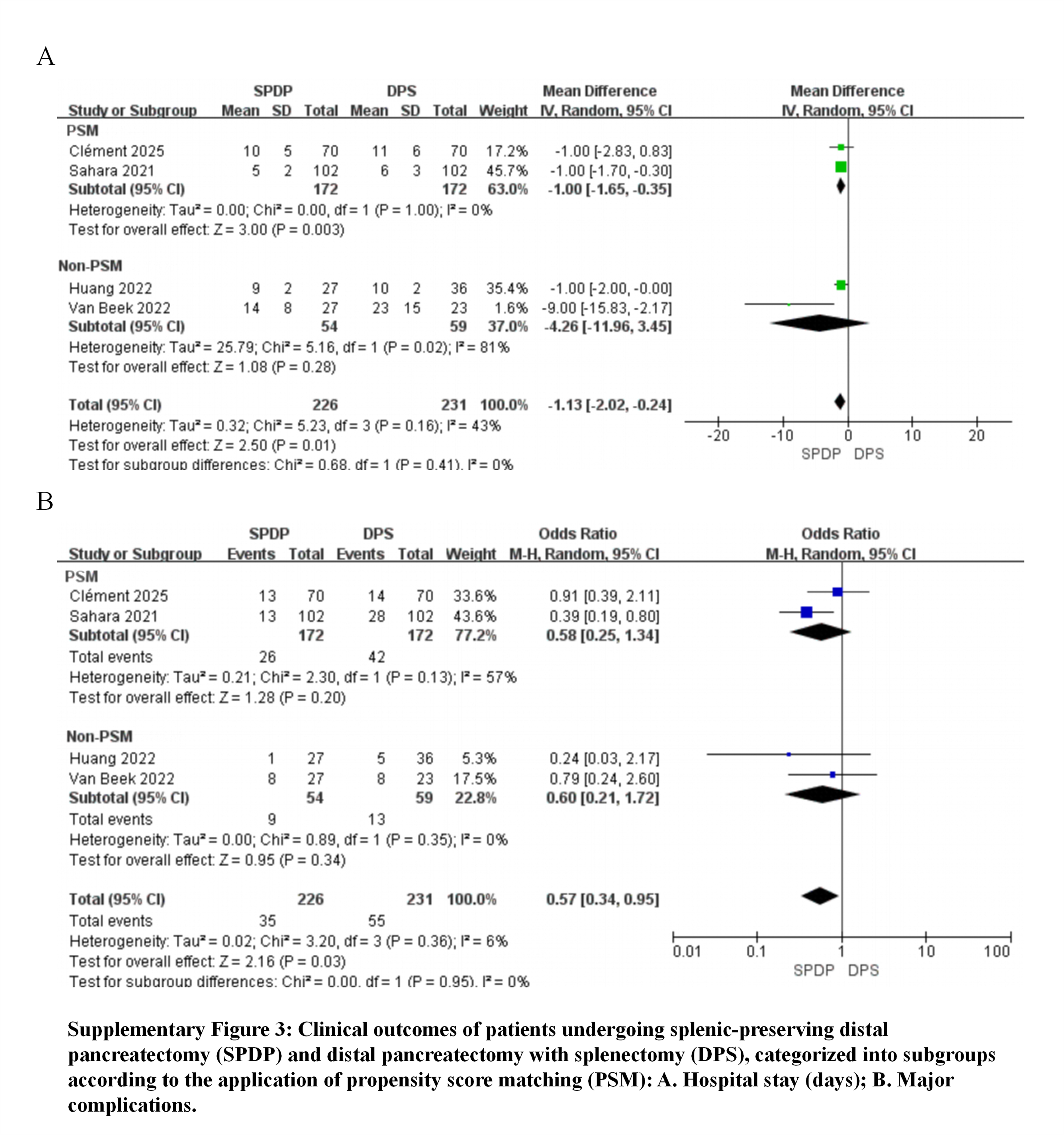

Supplement: Supplementary Figure 3 — Clinical outcomes of patients undergoing splenic-preserving distal pancreatectomy (SPDP) and distal pancreatectomy with splenectomy (DPS), categorized into subgroups according to the application of propensity score matching (PSM): (A) Hospital stay (days); (B) Major complications. [file Image3.tif]

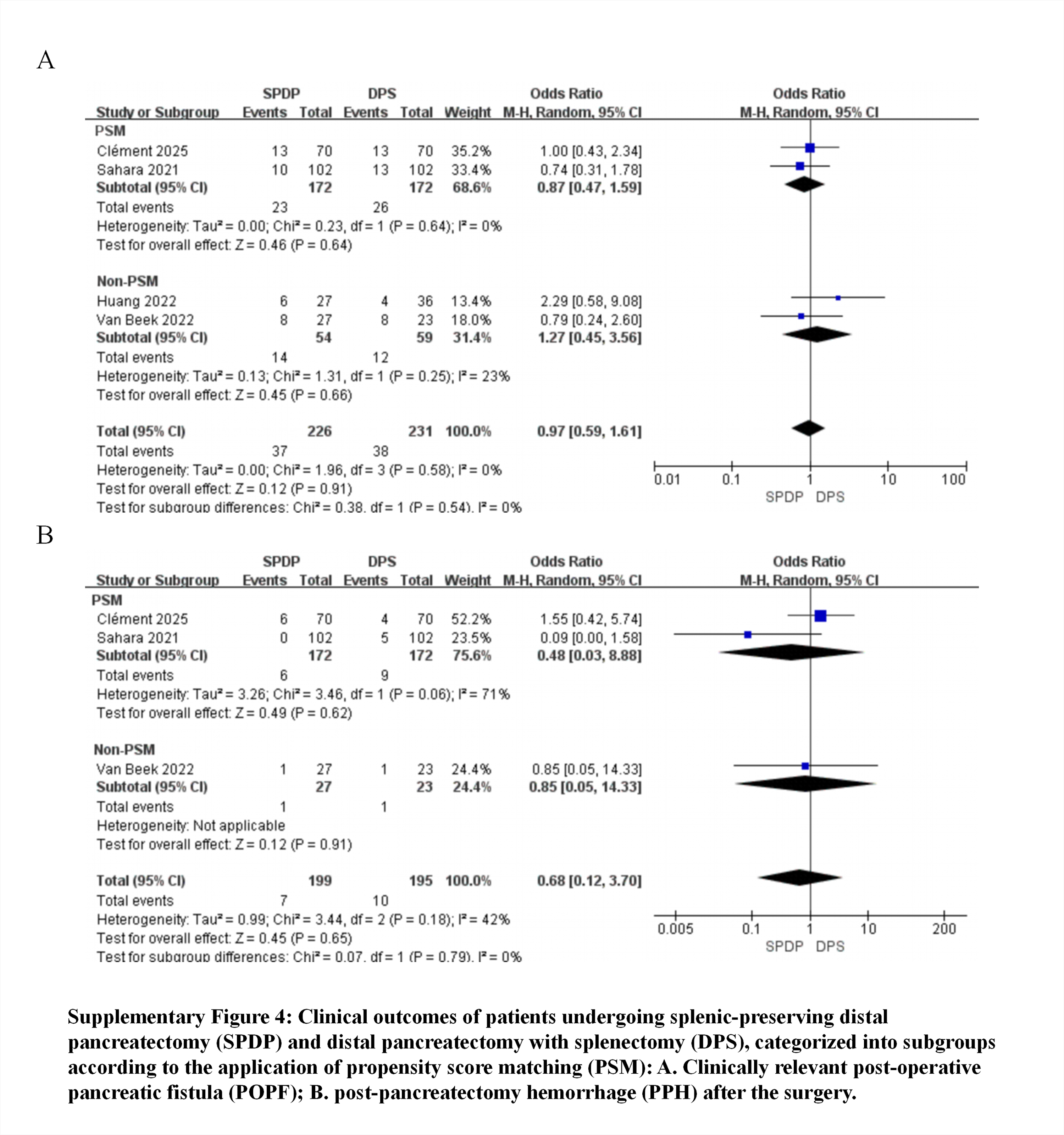

Supplement: Supplementary Figure 4 — Clinical outcomes of patients undergoing splenic-preserving distal pancreatectomy (SPDP) and distal pancreatectomy with splenectomy (DPS), categorized into subgroups according to the application of propensity score matching (PSM): (A) Clinically relevant post-operative pancreatic fistula (POPF); (B) post-pancreatectomy hemorrhage (PPH) after the surgery. [file Image4.tif]

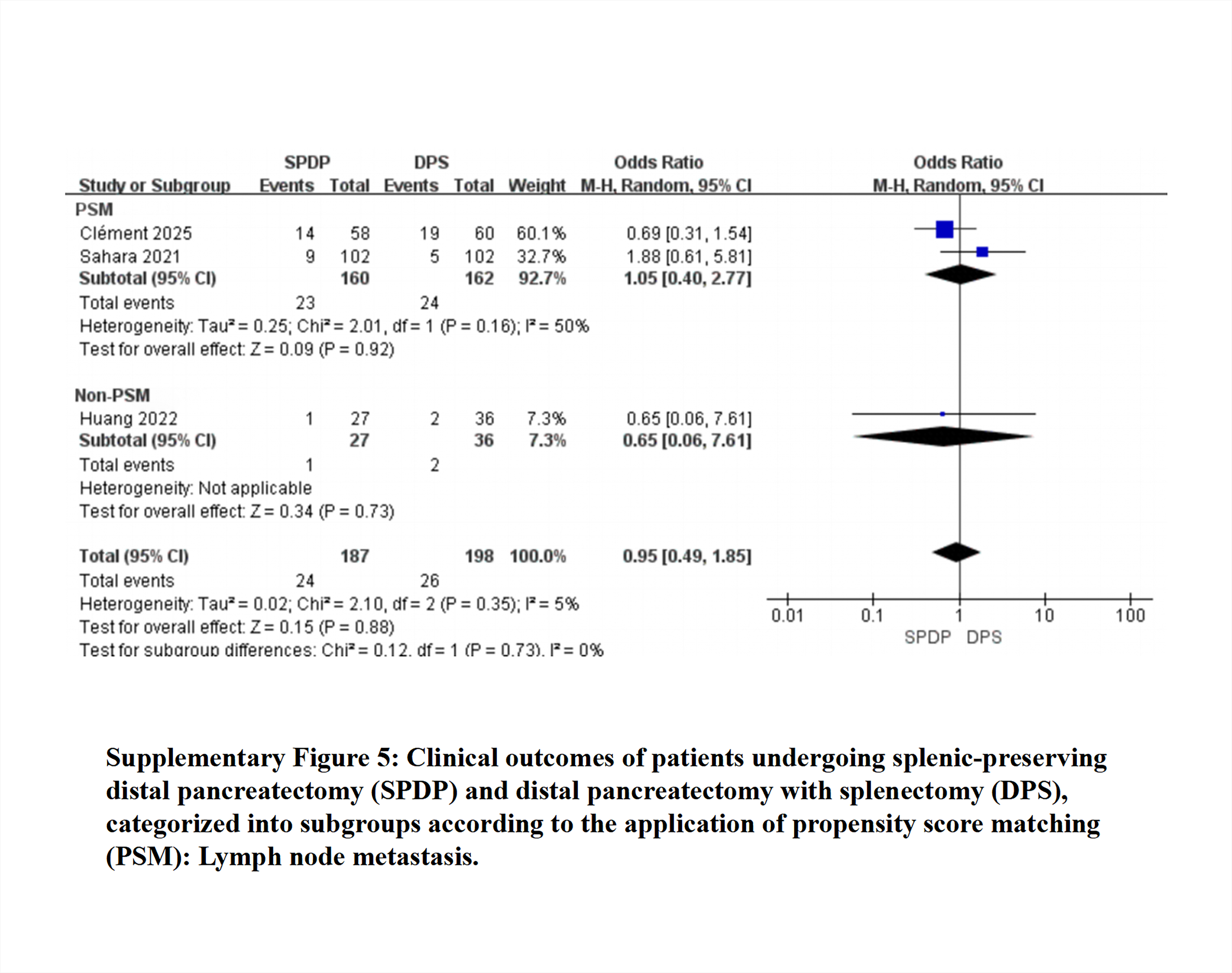

Supplement: Supplementary Figure 5 — Clinical outcomes of patients undergoing splenic-preserving distal pancreatectomy (SPDP) and distal pancreatectomy with splenectomy (DPS), categorized into subgroups according to the application of propensity score matching (PSM): Lymph node metastasis. [file Image5.tif]

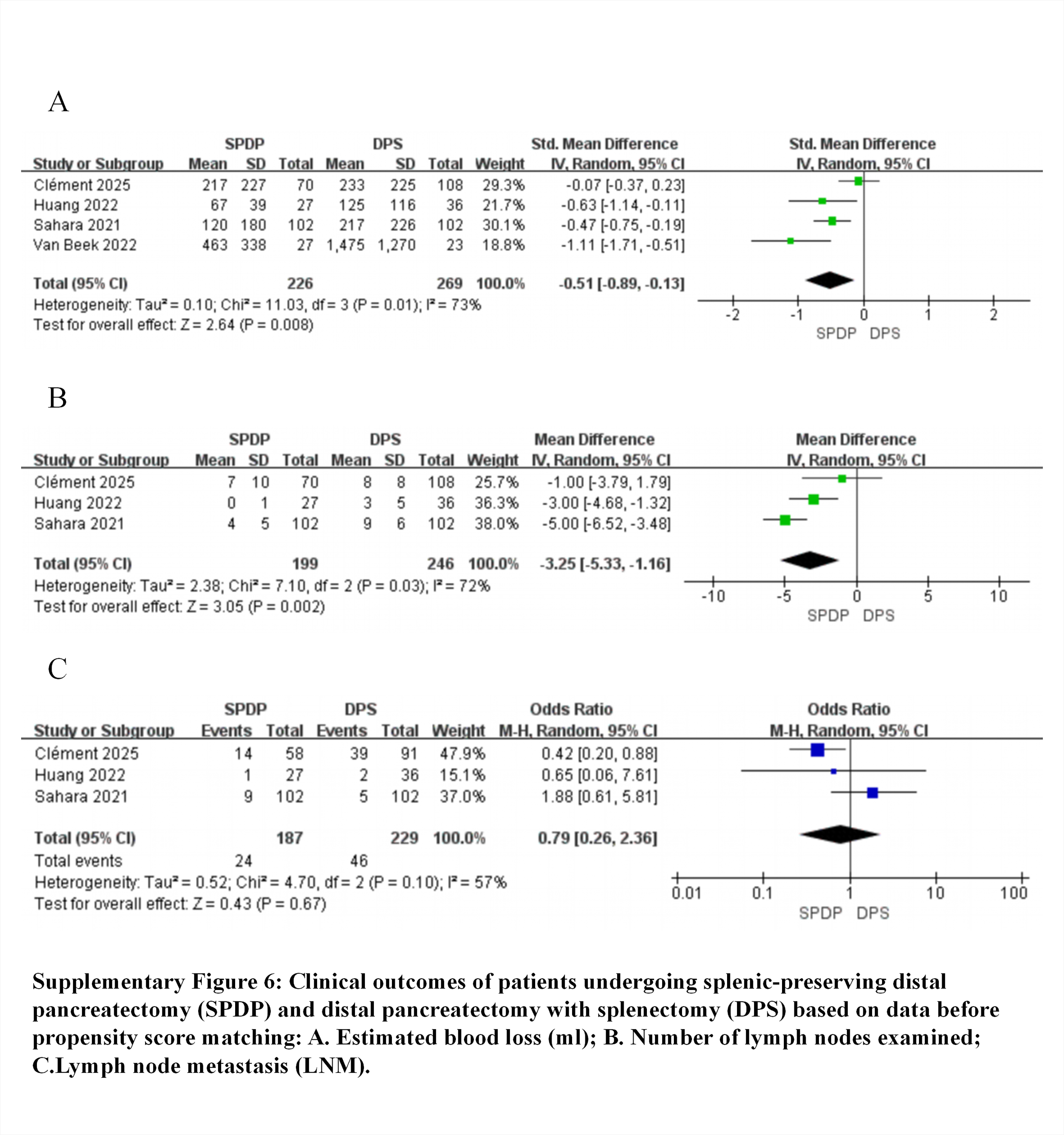

Supplement: Supplementary Figure 6 — Clinical outcomes of patients undergoing splenic-preserving distal pancreatectomy (SPDP) and distal pancreatectomy with splenectomy (DPS) based on data before propensity score matching: (A) Estimated blood loss (ml); (B) Number of lymph nodes examined; (C) Lymph node metastasis (LNM). [file Image6.tif]

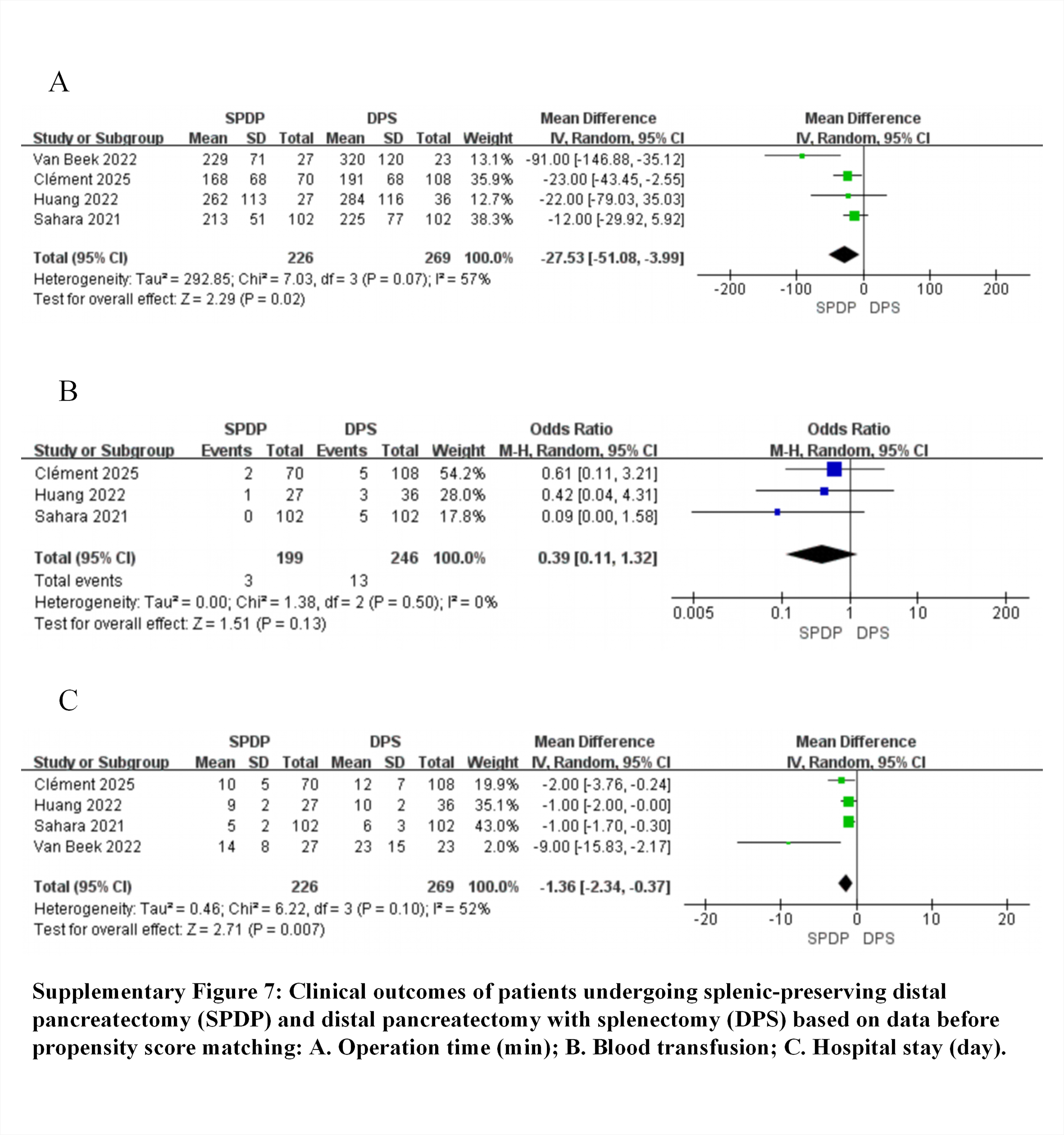

Supplement: Supplementary Figure 7 — Clinical outcomes of patients undergoing splenic-preserving distal pancreatectomy (SPDP) and distal pancreatectomy with splenectomy (DPS) based on data before propensity score matching: (A) Operation time (min); (B) Blood transfusion; (C) Hospital stay (day). [file Image7.tif]

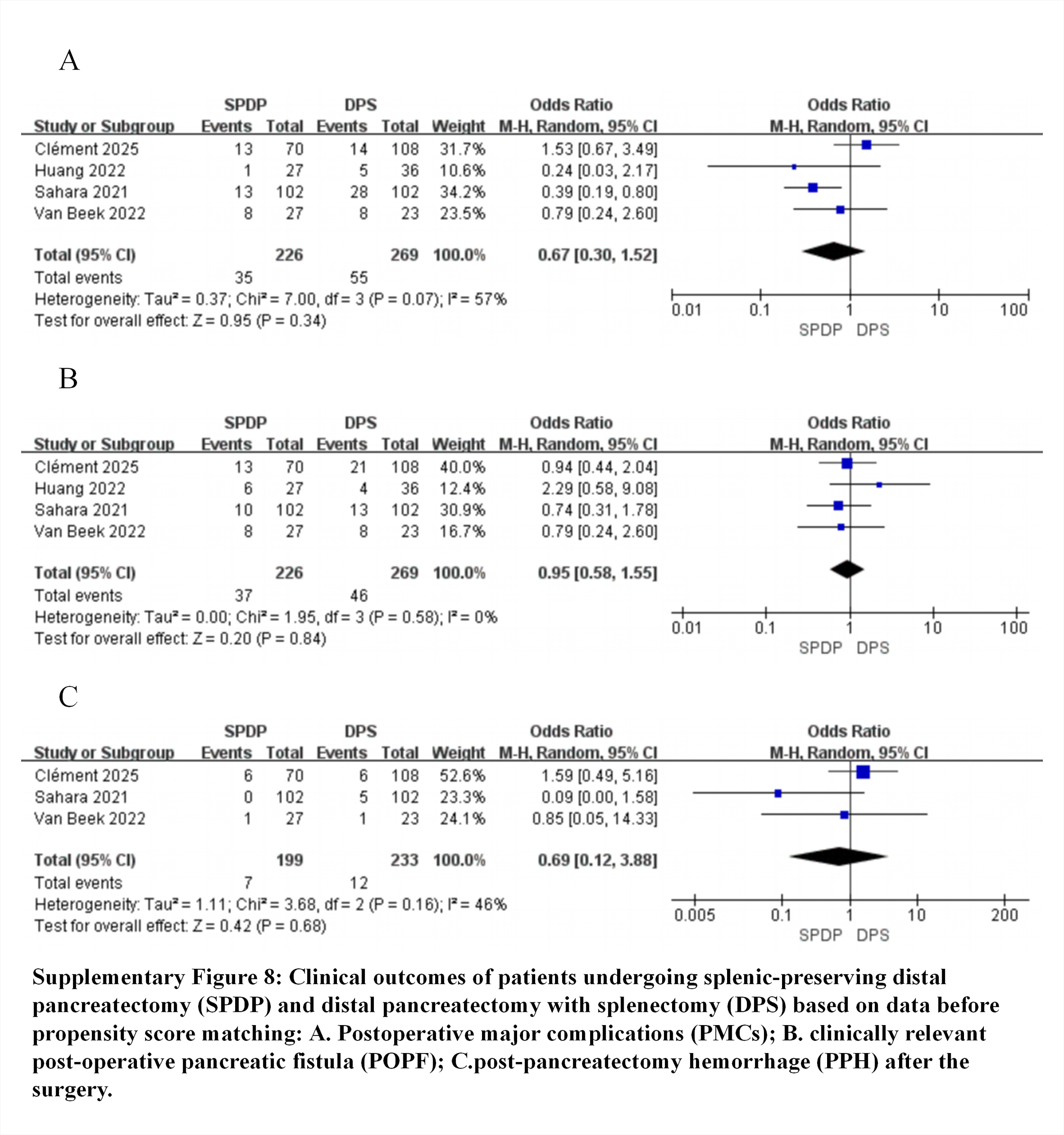

Supplement: Supplementary Figure 8 — Clinical outcomes of patients undergoing splenic-preserving distal pancreatectomy (SPDP) and distal pancreatectomy with splenectomy (DPS) based on data before propensity score matching: (A) Postoperative major complications (PMCs); (B) clinically relevant post-operative pancreatic fistula (POPF); (C) post-pancreatectomy hemorrhage (PPH) after the surgery. [file Image8.tif]
